# The mechanisms underlying the actions of Xuefu Zhuyu decoction pretreatment against neurological deficits after ischemic stroke in mice: The mediation of glymphatic function by aquaporin-4 and its anchoring proteins

**DOI:** 10.3389/fphar.2022.1053253

**Published:** 2022-12-13

**Authors:** Ting Yi, Ping Gao, Meng Hou, Huan Lv, Mengyuan Huang, Shanshan Gao, Jinrong He, Dongdong Yang, Weiyin Chen, Tianmin Zhu, Chang Yu, Fuyou Liu, Haiyan Yin, Shuoguo Jin

**Affiliations:** ^1^ School of Health and Rehabilitation, Chengdu University of Traditional Chinese Medicine, Chengdu, China; ^2^ Department of Neurology, Hospital of Chengdu University of Traditional Chinese Medicine, Chengdu, China; ^3^ School of Acupuncture and Tuina, Chengdu University of Traditional Chinese Medicine, Chengdu, China

**Keywords:** ischemic stroke, Xuefu Zhuyu decoction, glymphatic system, neuroprotection, aquaporin-4

## Abstract

Ischemic stroke (IS) has been associated with an impairment in glymphatic function. Xuefu Zhuyu Decoction (XFZYD) is widely used in the prevention and treatment of ischemic stroke. We hypothesized that Xuefu Zhuyu decoction pretreatment could attenuate early neurological deficits after ischemic stroke by enhancing the function of the glymphatic system. To prove our hypothesis, we carried out temporary middle cerebral artery occlusion and reperfusion surgery on C57BL/6 mice and then measured neurological score, infarct size and performed hematoxylin-eosin staining to assess stroke outcomes after 24 h of reperfusion. Subsequently, we injected fluorescent tracers in to the cisterna magna and evaluated tracer distribution in coronal brain sections. The polarization of aquaporin-4 (AQP4), colocalization of aquaporin-4, α-dystroglycan, β-dystroglycan and agrin were determined by immunofluorescence. Our research showed that pretreatment with Xuefu Zhuyu decoction significantly alleviated neurological scores, neurological deficits and pathological abnormalities in a mouse model of ischemic stroke. Importantly, Xuefu Zhuyu decoction pretreatment enhanced cerebrospinal fluid influx, protected aquaporin-4 depolarization and promoted the colocalization of aquaporin-4 with its anchoring proteins in the brain. Our findings highlight novel mechanisms underlying the neuroprotective effect of Xuefu Zhuyu decoction pretreatment on ischemic stroke-induced brain damage through the glymphatic system. Xuefu Zhuyu decoction pretreatment may offer a promising approach to slow the onset and progression of ischemic stroke.

## Introduction

Ischemic stroke (IS) is a globally significant neurological disease that is considered to be the leading cause of mortality and permanent disability; however, effective treatments remain limited (Krishnamurthi et al., 2020). Currently, recombinant tissue plasminogen activator (r-tPA) is the only thrombolytic agent approved by the Food and Drug Administration for the treatment of blood clots, but is only suitable for a small subset of patients due to the short therapeutic window (Catanese et al., 2017). Therefore, it is vital that we identify potential mechanisms and develop alternative treatments.

Mounting evidence indicates that traditional Chinese medicine can ameliorate dysfunction associated with post-ischemic stroke (Bu et al., 2020; Zhu et al., 2021). Notably, Xuefu Zhuyu decoction (XFZYD), a traditional Chinese prescription that is recorded in *Yilin Gaicuo*, contains eleven herbs (Table 1) and has been shown to reverse IS-induced neurological dysfunction (Lee et al., 2011; Shen et al., 2015; Xu et al., 2022). However, the specific mechanisms and potential targets of XFZYD against neurological deficits have yet to be investigated.

**TABLE 1:** Compositions of Xuefu Zhuyu decoction.

Plant name	Latin name	Chinese name	Medicinal part	Weigh(g)
*Prunus persica (L.) Batsch*	Semen Persicae	Tao Ren	Seed	12
*Carthamus tinctorius L*	Flos Carthami	Hong Hua	Flower	9
*Angelica sinensis (Oliv.) Diels*	Radix Angelicae Sinensis	Dang Gui	Root	9
*Rehmannia glutinosa Libosch*	Radix Rehmanniae	Di Huang	Root	9
*Achyranthes bidentata Blume*	Radix Achyranthis Bidentatae	Niu Xi	Root	9
*Paeonia lactiflora Pall*	Radix Paeoniae Rubra	Chi Shao	Root	6
*Citrus aurantium L*	Fructus Aurantii	Zhi Qiao	Fruit	6
*Glycyrrhiza uralensis Fisch*	Radix Glycyrrhizae	Gan Cao	Root	3
*Ligusticum chuanxiong*	Rhizoma Chuanxiong	Chuan Xiong	Root	5
*Platycodon grandiflorus (Jacq.) ADC.*	Radix Platycodonis	Jie Geng	Root	5
*Bupleurum chinense DC.*	Radix Bupleuri	Chai Hu	Root	3

The glymphatic system (GS) is a newly discovered system that clears waste from the central nervous system, promotes the exchange of cerebrospinal fluid (CSF) with interstitial fluid (ISF) to clean metabolic waste and brings new insights into the pathogenesis of neurological disorders, including IS (Iliff et al., 2012; Nedergaard, 2013; Rasmussen et al., 2018). Recent studies have provided insights into the role of glymphatic dysfunction in IS and indicated that the clearance of ISF outflow is reduced after ischemic infarct (Gaberel et al., 2014; Lin et al., 2020; Toh and Siow, 2021). Furthermore, research has demonstrated that dysfunction of the glymphatic system is accompanied by the significant accumulation of amyloid-beta (Aβ) and tau in stroke (Back et al., 2020; Lyu et al., 2021a; Lyu et al., 2021b). Therefore, it has been proposed that glymphatic dysfunction may act as a pathogenic mechanism underlying the onset and development of IS. Thus, improving glymphatic function and restoring glymphatic inflow represent novel therapeutic targets for IS (Lv et al., 2021).

Aquaporin-4 (AQP4) is essential for GS function and is densely localized in the astrocytic endfeet (Iliff et al., 2012). High polarization can reduce resistance for the bidirectional exchange of fluid and interstitial solutes in a manner that depends on specific anchoring mechanisms (Amiry-Moghaddam et al., 2004; Nagelhus and Ottersen, 2013; Mader and Brimberg, 2019). AQP4 is densely anchored to the perivascular basal lamina by the dystrophin-associated protein complex (DAPC) which consists of α-syntrophin, α-dystrobrevin, dystrophin, β-dystroglycan (β-DG) and α-dystroglycan (α-DG) (Nico et al., 2010; Nagelhus and Ottersen, 2013; Belhasan and Akaaboune, 2020). Agrin and laminin, components of the basal lamina, interact with α-DG to form a complex with β-DG that stretches through the plasma membrane (Hoddevik et al., 2020; Jorgačevski et al., 2020). Due to this complex molecular organization and close physical interactions, these water channels are unusually dense and positioned at the interface between the perivascular and interstitial spaces of the brain. Consequently, the localization and structure of the AQP4 channels can reduce the resistance to CSF-ISF exchange and maintain this delicate balance. Thus, AQP4 polarization and its colocalization with anchoring proteins in the astrocyte endfeet may serve as candidate mechanisms for disease intervention.

However, the association between XFZYD and GS in the treatment of stroke, and the potential mechanisms involved, have yet to be elucidated. In the present study, we investigated the role of XFZYD pretreatment on the neurological functions of a mouse model of temporary middle cerebral artery occlusion and reperfusion (tMCAO) and investigated the structural and molecular mechanisms underlying the neuroprotective effects of XFZYD pretreatment and the potential involvement of the GS, particularly with regards to the role of AQP4 polarization and the co-localization of anchoring proteins.

## Materials and methods

### Preparation of Xuefu Zhuyu decoction

XFZYD contains 11 herbs, as shown in Table 1. We purchased XFZYD from the Pharmacy of the Hospital of Chengdu University of Traditional Chinese Medicine; further processing was performed by the pharmacy department. According to the standard process, all herbs were soaked with an 8-fold volume of water for 60 min and decocted for 40 min; this process was repeated twice for extraction. After filtration, the filtered decoctions were mixed together and concentrated to 1 g/ml with a rotary evaporator.

### Animals

Specific pathogen-free male C57BL/6 mice, weighing 20–25 g and aged 8–12 weeks were purchased from Gempharmatech Co., Ltd. (Chengdu, China, license number: SCXK (Chuan) 2020-034) and maintained at a temperature of 20°C–26°C with a 12 h light/dark cycle and free access to food and water. The experimental protocol in this study was approved by the Ethics Committee of Chengdu University of Traditional Chinese Medicine (Reference: 2022-04). All mice were allowed to adapt to these environmental conditions for 1 week prior to experimentation.

### Experimental design

**FIGURE 1:**
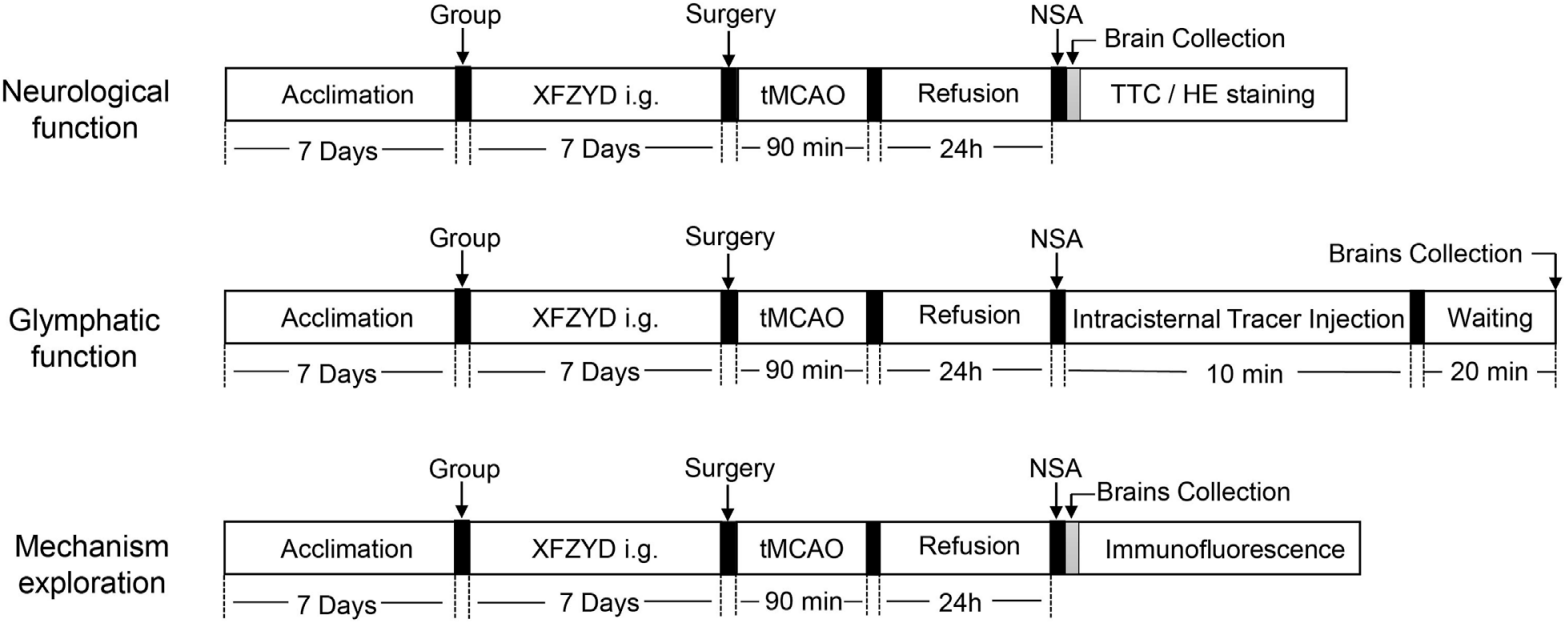
Schematic diagram depicting the entire study. Three experiments were carried out; these were designed to investigate the effect of XFZYD pretreatment on neurological function, glymphatic function and identify the potential mechanisms involved. XFZYD, Xuefu Zhuyu decoction; tMCAO, temporary middle cerebral artery occlusion and reperfusion; NSA, neurological score assessment; TTC, 2,3,5-triphenyltetrazolium chloride; HE, Hematoxylin-Eosin.

In this study, there were three major aims. Experiment 1 investigated the effects of XFZYD pretreatment on functional recovery after stroke surgery. Experiment 2 investigated whether the effect of XFZYD pretreatment on neurological function was related to GS. Experiment 3 further explored the mechanisms underlying the effects observed in Experiment 1 and Experiment 2. In brief, mice were randomly divided into three groups. The sham group (n = 24) was given normal saline for 7 days then under sham experiments. Mice in the model group (tMCAO, n = 42) were intragastrically administered the same amount of saline for 7 days before stroke experiments. Mice in the positive group (tMCAO + XFZYD pretreatment, n = 42) were intragastrically administered with 10 times of the dosage commonly used to human adults in clinics (the medium dose, 15 ml/kg/d) twice a day for seven consecutive days prior to surgery. Animals underwent neurological assessment 24 h after reperfusion; then, their brains were collected for a series of experiments. Six mice per group for 2,3,5-triphenyltetrazolium chloride (TTC) staining and six mice per group for hematoxylin-eosin (HE) staining and six mice per group for immunofluorescence staining. The remaining mice were used for intracisternal tracer injections. Figure 1 shows a flow diagram depicting the study design.

### Induction of tMCAO injury in mice

Mice were fasted for 8 h before surgery. Mice were anesthetized with 1% pentobarbital (50–70 mg/kg, intraperitoneal injection) and tMCAO surgery was performed as described previously (Longa et al., 1989). In brief, the left common carotid artery (CCA), external carotid artery (ECA) and internal carotid artery (ICA) were carefully exposed and isolated. A nylon filament was inserted into the ECA and then passed through the ICA and advanced to occlude the origin of the middle cerebral artery (MCA) for 90 min. Body core temperature was maintained at 37°C with a heating blanket throughout this process. Then, 90 min after the occlusion, the mice were re-anesthetized and the occluding filament was removed to allow 24 h of blood flow. Sham-operated animals underwent the same anesthesia and exposure of the arteries, but the filament was not inserted.

### Evaluation of neurologic deficits

After 24 h of reperfusion, neurological tests were performed by an investigator who was blinded to the experimental protocol, as described previously (Longa et al., 1989). Neurological function was scored on a 4-point scale: (0) = no deficits; (1) = difficulty in fully extending the contralateral front paw; (2) = circling to the contralateral side; (3) = falling to the opposite side, and (4) = no consciousness or ambulation. Mice with scores of one to three were included in the follow-up experiments while those with scores of 0 or 4 were excluded.

### Quantification of brain infarct volume

The volume of brain infarction was evaluated by TTC staining after 24 h of reperfusion and neurological examination. Subsequently, the brains were quickly removed on ice and then frozen at −20°C for 10 min. Then, the tissues were cut into 1.5-mm-thick coronal sections and then incubated with 2% TTC solution (Solarbio, China) at 37°C for 15 min. The sections were turned over every 5 min in the dark and photographed after fixation in 4% paraformaldehyde (PFA) for 1 h. The infarcted area was then measured and analyzed by Image J (NIH, United States). Infarct volume = (infarct volume on the infarct side/total brain volume) × 100%.

### Histopathological examination

HE staining was conducted according to previous protocols (Jin et al., 2019). In brief, the brain tissues were embedded in paraffin wax after perfusion-fixation and fixed with 4% PFA overnight and cut coronally into 5-μm sections which were then stained with hematoxylin and eosin.

### Intracisternal tracer injections

Intracisternal tracer injections were performed as described previously (Iliff et al., 2012). The fluorescent CSF tracer (fluorescein isothiocyanate-conjugated dextran, FITC-D3, 3 kDa, Invitrogen, United States) was diluted in artificial CSF at a concentration of 0.5% (w/v). Each mouse was anesthetized and the head was secured into a stereotaxic frame; then, we exposed the atlantooccipital membrane of the cisterna magna (CM) by surgery and attached a 30-gauge needle to the tracer-filled PE10 tube which was then carefully inserted into the CM with a Hamilton syringe pump (KD Scientific, United States). The intracisternal injection was carried out at a rate of 1 μL/min for 10 min. After injection, the needle was left in place for at least 5 min to prevent backflow. Mice were then placed on a heating pad until 20 min post-injection. Brains were fixed overnight in 4% PFA, cut into coronal frozen slices (10 μm) and then imaged. Upon acquisition, tracer penetration was quantified by two sets of blinded investigators using Image J.

### Immunofluorescence

Brains were removed, post-fixed in 4% PFA overnight, dehydrated in 20%–30% sucrose and then sectioned consecutively with a frozen microtome (Leica, Germany). Coronal brain sections (10 μm) were immunostained for glial fibrillary acidic protein (GFAP), AQP4, α-DG, β-DG and agrin and double stained with GFAP/AQP4 for AQP4 polarization, AQP4/α-DG, AQP4/β-DG and AQP4/agrin for the colocalization of AQP4 with anchoring proteins. The immunofluorescence procedure was carried out as reported previously (Da Mesquita et al., 2021). Sections were rinsed in PBS and blocked with 5% bovine serum albumin (BSA; Solarbio, China) for 30 min. Subsequently, the sections were incubated with appropriate dilutions of primary antibodies overnight: mouse anti-GFAP (1: 500, Servicebio, China); rabbit anti-AQP4 (1: 400, Servicebio, China); mouse anti-α-DG (1:100, Santa Cruz, United States); mouse anti-β-DG (1:100, Santa Cruz, United States), and mouse anti-agrin (1:100, Santa Cruz, United States). The following morning, appropriate secondary antibodies (488-conjugated goat anti-rabbit (1:400; Servicebio, China) and cy3-conjugated goat anti-mouse (1:300; Servicebio, China)) were incubated for 1 h at room temperature in the dark. After washing and mounting, sections were observed with a fluorescent microscope (Nikon, Japan). The researcher analyzing the images and data was blinded to the experimental conditions.

### Aquaporin-4 polarization analysis

Images were acquired at a size of 700 × 700 µm in the corpus callosum (CC) and analyzed by FIJI software. Each 700 × 700 µm image was divided into 25 sub-regions that were 140 × 140 µm in size and 20% of the sub-regions were randomly selected to evaluate AQP4 polarity distribution to represent the global situation. The red channel was GFAP, the green channel was AQP4, and the blue channel was DAPI. Based on previous studies (Zeppenfeld et al., 2017; Cao et al., 2022), DAPI and GFAP immunostaining surrounded the voids as capillaries. Then, we drew a region of interest (ROI) within 3 µm from the capillary; the green fluorescence in this area represented the fluorescence intensity of AQP4 in the perivascular space. The fluorescence intensity of the corresponding subregion represented the global expression of AQP4. Thus, the AQP4 polarization index was calculated by the ratio of the mean fluorescent intensity of AQP4 around the perivascular space to its global distribution. Three to six vessels were assessed in each subregion per brain section.

### Colocalization of aquaporin-4 with α-DG, β-DG and agrin analysis

Based on previous studies (Zinchuk and Grossenbacher-Zinchuk, 2009; Dunn et al., 2011), we applied Pearson’s correlation coefficient and Manders’ overlap colocalization coefficient to measure colocalization in fluorescence microscope images, which were applied in image analysis software packages and implemented in ImageJ *via* the coloc 2 plugin. Manders’ tM1 and Manders’ tM2 were used to represent the ratio of colocalization for different fluorophores. Values above 0.5 indicated colocalization. We used these metrics to measure the colocalization of AQP4, α-DG and β-DG in the meningeal.

### Statistical analysis

All data were statistically analyzed by one-way analysis of variance (ANOVA) when data were normally distributed. For non-normally distributed data, further Mann–Whitney *U* test was used. The significant differences were set at *p* < 0.05 (SPSS 26.0 software, Armonk, NY, United States; Prism 8, GraphPad, La Jolla, CA, United States). Data were expressed as the mean ± standard deviation (SD) if normally distributed; otherwise, data not normally distributed were expressed as medians and interquartile ranges.

## Results

### Xuefu Zhuyu decoction pretreatment alleviated neurological function after tMCAO in mice

**FIGURE 2:**
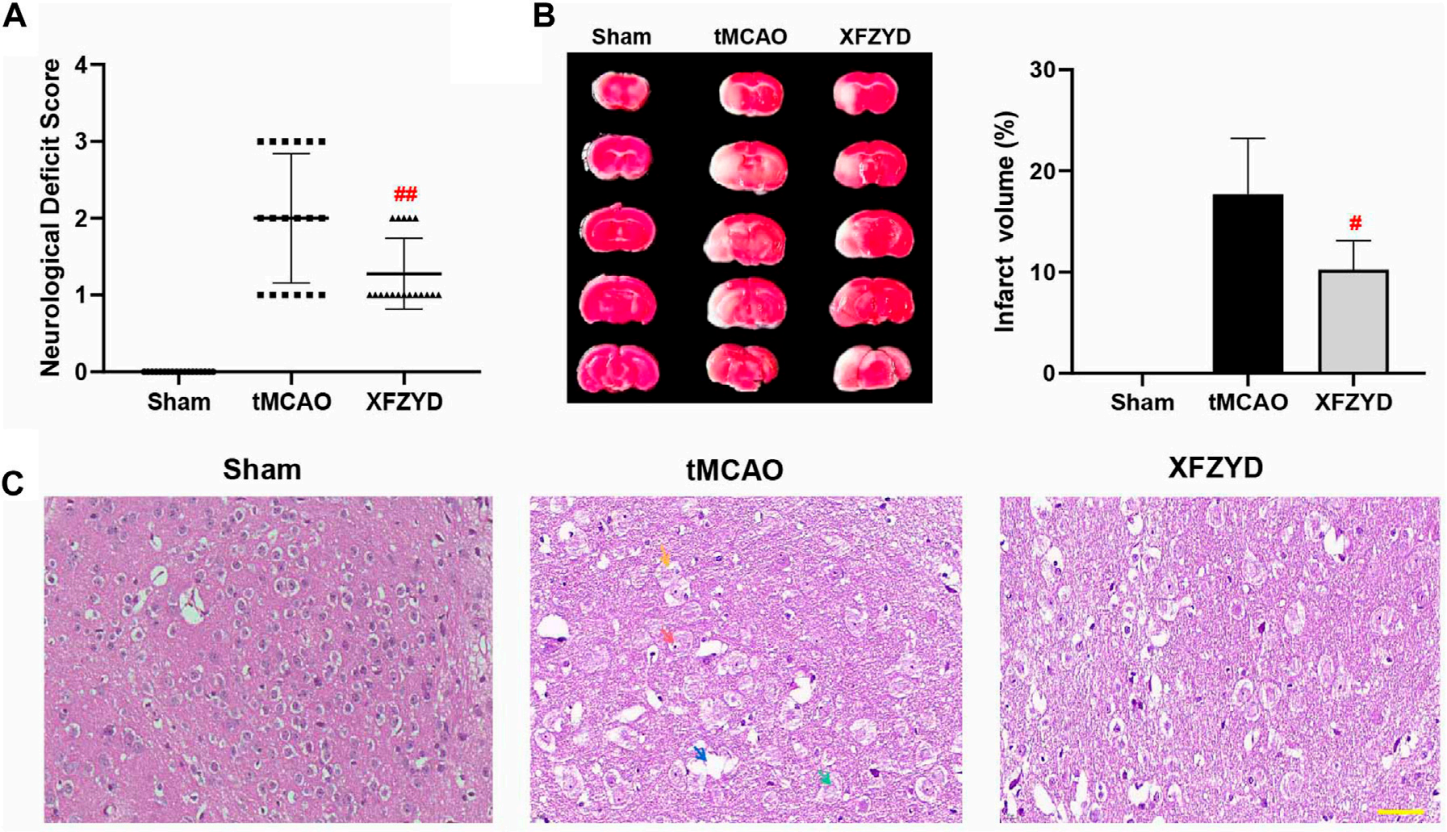
XFZYD pretreatment promoted neurological protection after tMCAO surgery. **(A–B)** Neurological deficit scores and TTC staining were measured 24 h after surgery. **(A)** Comparison of neurological deficit scores in tMCAO and XFZYD groups (n = 18 in each group, ^##^ *p* < 0.01 vs. the tMCAO group). **(B)** Representative brain sections stained with TTC are shown and infarct volume was quantified and compared between the XFZYD and tMCAO groups (n = 6 in each group, ^#^ *p* < 0.05 vs. the tMCAO group). **(C)** Representative photomicrographs of HE staining (Scale bar, 50 μm). Nerve cells in the infarcted area had incurred damaged, as demonstrated by nucleolus pyknosis (red arrow), fragmentation (yellow arrow), nucleolus dissolution (green arrow) and vacuolated spaces (blue arrow).

One hundred and eight mice were distributed into three groups. The success rate of the tMCAO group was 59.5% (25 of 42) while that of the XFZYD group was 61.9% (26 of 42). At 24 h post-surgery, neurological function was assessed by an observer who was blinded to the experimental conditions. There was no neurological deficit in the sham group. However, both the tMCAO and XFZYD groups appeared to present with different degrees of motor behavioral impairments in the contralateral limbs. Pretreatment with XFZYD before tMCAO significantly reduced the neurological deficit scores when compared with tMCAO group (*p* < 0.01, Figure 2A and Table 2).

**TABLE 2:** Comparison of neurological deficit scores in tMCAO and XFZYD groups.

Groups	n	M(P_25_,P_75_)	Z	*p*
tMCAO	18	2 (1,3)	2.724	0.006
XFZYD	18	1 (1,2)		

We assessed brain infarct volume by TTC staining 24 h after reperfusion. The volumes of cerebral infarctions are shown in Figure 2B. We found that the sham group had no infarction or edema formation. The left brain tissue of mice in the tMCAO group and XFZYD group were obviously swollen. White infarcts in the cerebral cortex and striatum were clearly visible by TTC staining. However, pretreatment with XFZYD significantly attenuated these neurological deficits (*p* < 0.05, Figure 2B).

HE staining is shown in Figure 2C; no obvious neuronal damage was observed in the sham group in which the cell structure was complete. Following tMCAO injury, nerve cells in the infarcted area were damaged and exhibited nucleolus pyknosis (red arrow), fragmentation (yellow arrow), nucleolus dissolution (green arrow) and vacuolated spaces (blue arrow). However, the pathological abnormalities were significantly reduced following pretreatment with XFZYD when compared to the model group.

### Glymphatic influx was damaged after tMCAO but rescued by Xuefu Zhuyu decoction pretreatment

**FIGURE 3:**
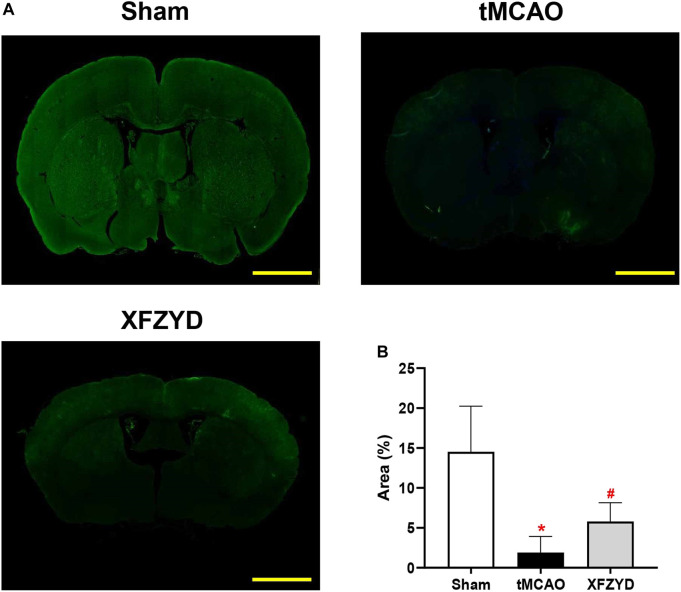
The function of the glymphatic pathway was impaired after tMCAO but rescued by pretreatment with XFZYD. **(A)** Representative images of coronal brain sections showing FITC-D3 distribution within the brain at 30 min (Scale bar, 1 mm). **(B)** Quantitative analysis of the fluorescence intensity and penetration area of data A (n = 4 mice per group, *p < 0.05 vs. the sham group, ^#^ *p* < 0.05 vs. the tMCAO group). CSF, Cerebrospinal fluid; FITC-D3, fluorescein isothiocyanate-conjugated dextran.

To ascertain the function of the GS in mice, we injected FITC-D3 into the CSF pool of CM and examining fluorescent tracer penetration into the brain. Representative images at 30 min are shown in Figure 3A. The parenchymal distribution of the green fluorescent tracer represented glymphatic influx. It is important to note that FITC-D3 influx was significantly decreased in the tMCAO group when compared to the sham group (*p* < 0.05, Figure 3B). When compared with the tMCAO group, there was a trend for increased tracer distribution following XFZYD pretreatment (*p* < 0.05, Figure 3B).

### Xuefu Zhuyu decoction pretreatment alleviated the aquaporin-4 depolarization induced by tMCAO injury

**FIGURE 4:**
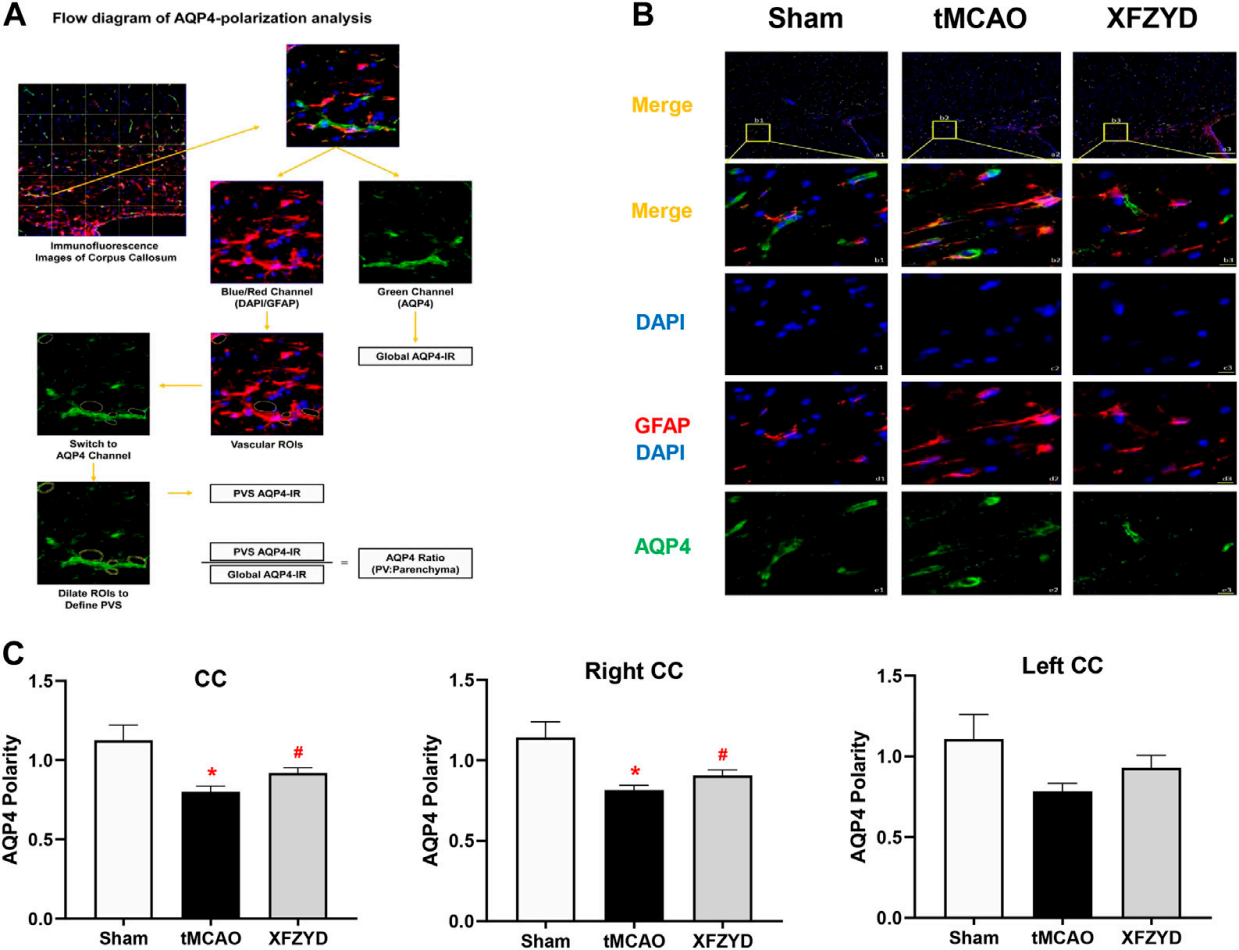
XFZYD restored AQP4 depolarization after tMCAO. **(A)** Flow diagram of AQP4-polarization analysis. **(B)** Representative images of AQP4 polarity in the CC. (a1-a3) Representative images showing the polarization of AQP4, GFAP and DAPI in different groups (green, AQP4; red, GFAP; blue, DAPI; scale bar, 200 μm). (b1-b3) Enlarged views of a1-a3 (scale bar, 10 μm). (c1-c3) The same regions but showing DAPI (scale bar, 10 μm). (d1-d3) Same regions as for GFAP and DAPI but showing vascular ROIS (scale bar, 10 μm). (e1-e3) AQP4 channels (scale bar, 10 μm). **(C)** Quantitative analysis of AQP4 polarization in the whole CC, the left CC and right CC (^*^ *p* < 0.05 vs. the sham group, ^#^ *p* < 0.05 vs. the tMCAO group; n = 15 in CC from six mice per group). CC, corpus callosum.

AQP4 polarity plays a significant role in GS and localizes to the endfeet membrane of the perivascular space abutting the basal lamina to promote CSF-ISF exchange. To gain insights into whether AQP4 was responsible for the mechanistic action of XFZYD pretreatment in improving glymphatic function, we investigated AQP4 expression and localization with GFAP and DAPI to analyze AQP4 polarization in the CC (Figure 4A). Representative images of AQP4 polarization in the CC are shown in Figure 4B. Immunofluorescence findings showed that AQP4 channels were observed within the astrocytic somata and not in the astrocytic endfeet in the tMCAO group (Figure 4B). However, in the XFZYD group, AQP4 was highly polarized and most AQP4 immunoreactivity was confined to the perivascular area (Figure 4B). Analysis showed that AQP4 polarity in the right CC and all of the CC was significantly higher in the XFZYD group than in the tMCAO group (*p* < 0.05, n = 6, Figure 4C).

### Xuefu Zhuyu decoction pretreatment promoted the colocalization of aquaporin-4 with α-DG, β-DG and agrin after tMCAO in mice

**FIGURE 5:**
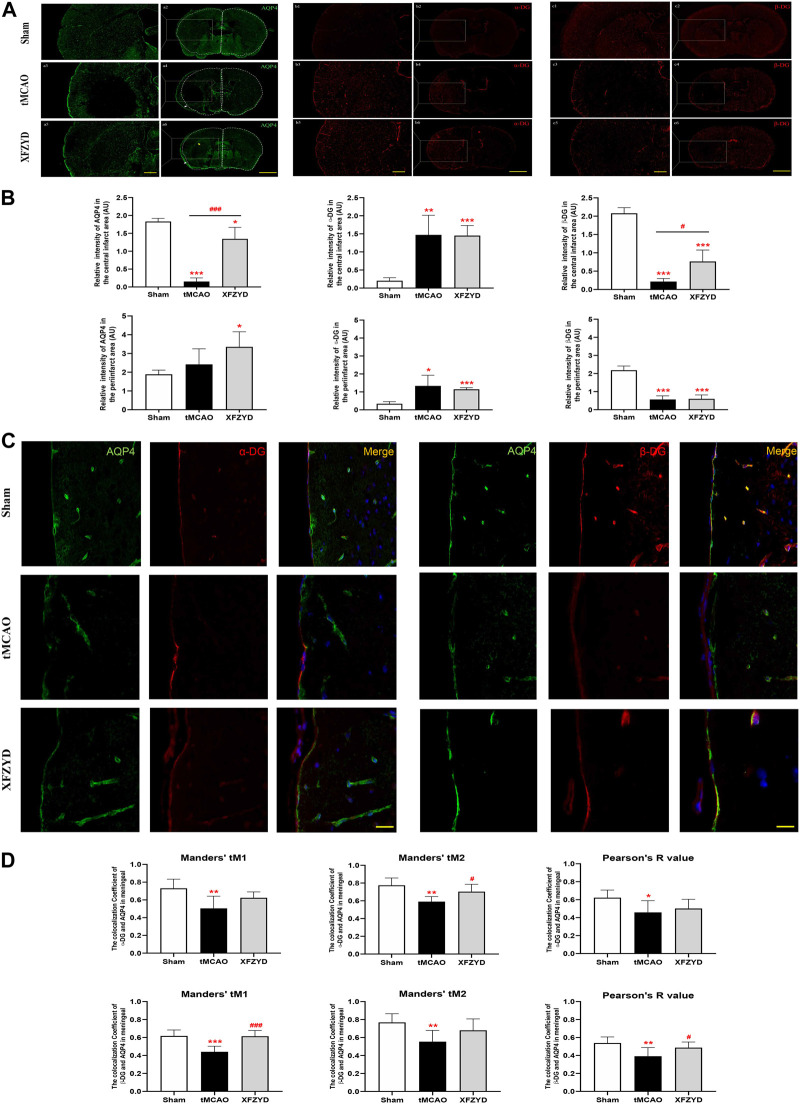
The effects of tMCAO and XFZYD on the colocalization of AQP4, α-DG and β-DG. **(A)** The immunostaining of AQP4, α-DG and β-DG in the infarct and periinfarct areas (Scale bars, 200μm and 500 μm). **(B)** Quantitative analysis of data A in infarct area and periinfarct area. **(C)** Representative colocalization images of AQP4, α-DG and β-DG immunostaining in the meningeal (α-DG, left; β-DG, right, Scale bar, 10 μm). **(D)** Quantification of the colocalization of AQP4, α-DG and β-DG by Manders’ tM1, Manders’ tM2 and Pearson’s R value in different groups. (^*^ *p* < 0.05, ^**^ *p* < 0.01, ^***^ *p* < 0.001 vs. the sham group, ^#^ *p* < 0.05, ^###^ *p* < 0.001 vs. the tMCAO group; Data represent mean ± SD, n = 6).

α-DG binds to AQP4 in astrocytes, whereas β-DG is a membrane-spanning protein that links α-DG to the cytoskeleton and other intracellular components (Jorgačevski et al., 2020). Since we demonstrated that XFZYD alleviated AQP4 depolarization after tMCAO, we further hypothesized that there may be a potential relationship between AQP4, α-DG and β-DG. To verify this hypothesis, we investigated the expression of AQP4, α-DG and β-DG in the brains of experimental mice. As shown in Figure 5A, there were clear differences in immunofluorescence between the infarct and peri-infarct areas. The fluorescence intensity of AQP4 in the infarct and peri-infarct areas of the XFZYD group (*p* < 0.05, n = 6, Figure 5B) and infarct area of the tMCAO group (*p* < 0.001, Figure 5B) were both significantly lower when compared with the sham group. In the XFZYD group, AQP4 fluorescence intensity in the infarct area was significantly higher than in the tMCAO group (*p* < 0.001, Figure 5B). Similarly, when compared with the sham group, the fluorescence intensity of α-DG was also significantly higher in the tMCAO group in both the infarct area (*p* < 0.01, Figure 5B) and the peri-infarct area (*p* < 0.05, Figure 5B). XFZYD group from infarct and periinfarct area was both higher than the control group (*p* < 0.001, Figure 5B). In addition, the fluorescence intensity of β-DG in the infarct and peri-infarct areas were both significantly lower in the tMCAO group and XFZYD group (*p* < 0.001, Figure 5B) when compared with the sham group. In the XFZYD group, β-DG fluorescence intensity in the infarct area was significantly higher than in the tMCAO group (*p* < 0.05, Figure 5B).

To further investigate the relationship between AQP4 and α-DG, as well as between AQP4 and β-DG, we used Pearson’s correlation coefficient and the Manders’ overlap coefficient to analyze colocalization. Representative images of colocalization between AQP4 and α-DG (Figure 5C, left) and between AQP4 and β-DG (Figure 5C, right) are shown. Immunofluorescence assays showed that α-DG and β-DG were expressed smoothly around the vascular wall in mouse brains without tMCAO. In the meningeal, the colocalization of AQP4, α-DG and β-DG in the tMCAO group were significantly lower than in the sham group, both in terms of Pearson’s R value (α-DG, *p* < 0.05; β-DG, *p* < 0.01), Manders’ tM1 (α-DG, *p* < 0.01; β-DG, *p* < 0.001) and Manders’ tM2 (*p* < 0.01). Pretreatment with XFZYD rescued the colocalization of AQP4, α-DG and β-DG when compared with the tMCAO group (Manders’ tM2, α-DG, *p* < 0.05; Manders’ tM1, β-DG, *p* < 0.001; Pearson’s R value, β-DG, *p* < 0.05).

**FIGURE 6:**
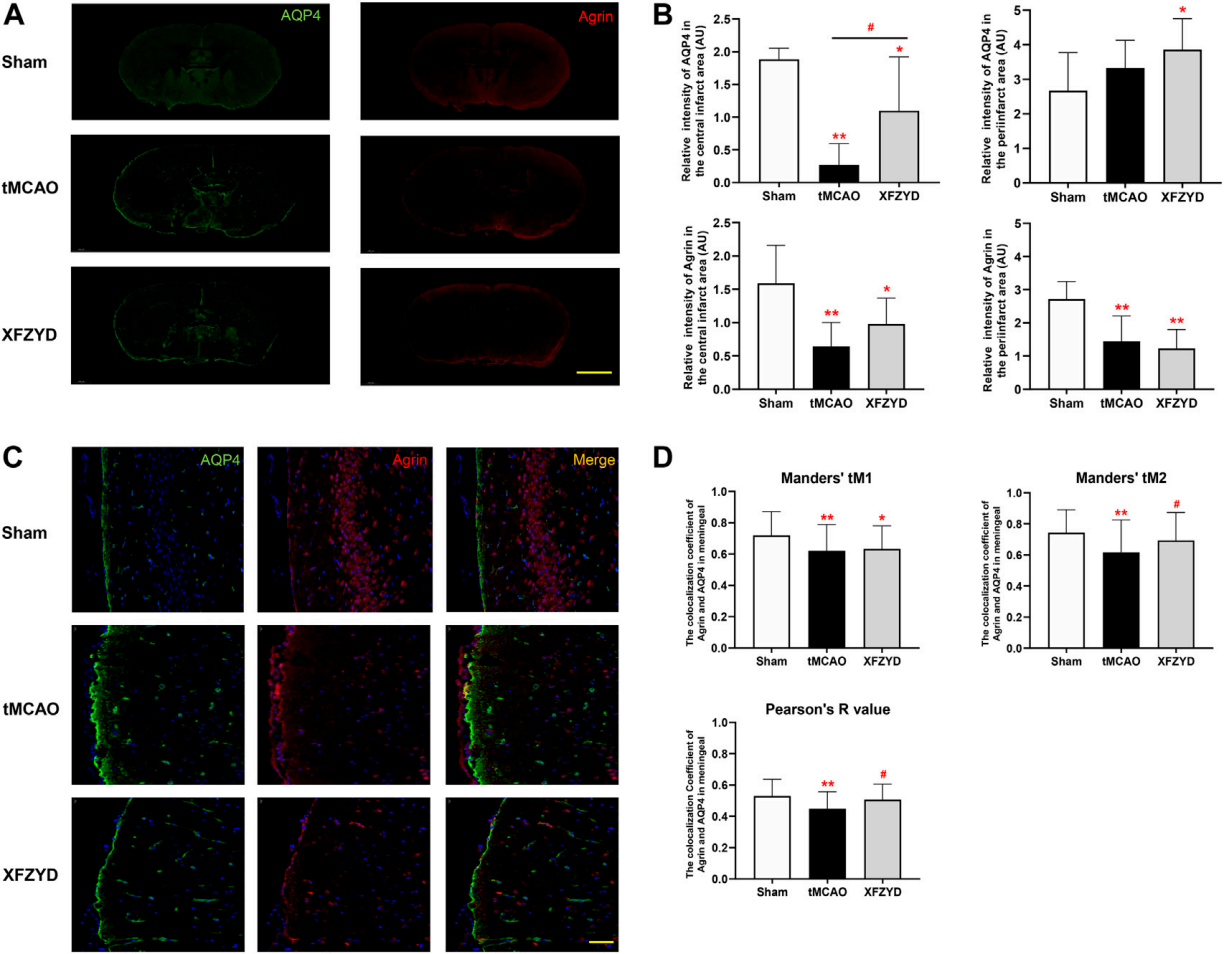
Effect of tMCAO and XFZYD on the colocalization of AQP4 and agrin. **(A)** The immunostaining of AQP4 and agrin in the three groups (Scale bar, 1 mm). **(B)** Quantitative analysis of data A in the infarct and periinfarct areas. **(C)** Representative colocalization images of agrin and AQP4 immunostaining in the meningeal (Scale bar, 20 μm) **(D)** Quantification of the colocalization of AQP4 and agrin by Manders’ tM1, Manders’ tM2 and Pearson’s R value from different groups in the meningeal (*p < 0.05, **p < 0.01 vs. the sham group, ^#^ *p* < 0.05 vs. the tMCAO group; Data represent mean ± SD, n = 6).

The relationship between agrin and AQP4 is also an important determinant for astrocyte polarity. Therefore, we also investigated the expression and localization of AQP4 and agrin. Whole brain immunofluorescence showed that the intensity of agrin in the tMCAO group and the XFZYD group was reduced both in the infarct and peri-infarct area (Figures 6A,B). In the central infarct area, the intensity of AQP4 and agrin in the tMCAO group and the XFZYD group were both lower than control group (tMCAO, *p* < 0.01; XFZYD, *p* < 0.05). In the peri-infarct area, the intensity of AQP4 in the XFZYD group was higher than the control group (*p* < 0.05), while agrin in the tMCAO group and the XFZYD group were both lower than control group (*p* < 0.01). Representative images for the colocalization of AQP4 and agrin in the meningeal were shown in Figure 6C, which was significantly different between groups in terms of immunofluorescence, as determined by Pearson’s correlation coefficient and Manders’ overlap colocalization coefficient (Figure 6D). In terms of Manders’ tM1, the sham group was significantly higher than the tMCAO group (*p* < 0.01) and XFZYD group (*p* < 0.05). With regards to Manders’ tM2 and the Pearson’s R value, the sham group was also significantly higher than the tMCAO group (*p* < 0.01) and the XFZYD group was significantly higher than the tMCAO group (*p* < 0.05).

## Discussion

**FIGURE 7:**
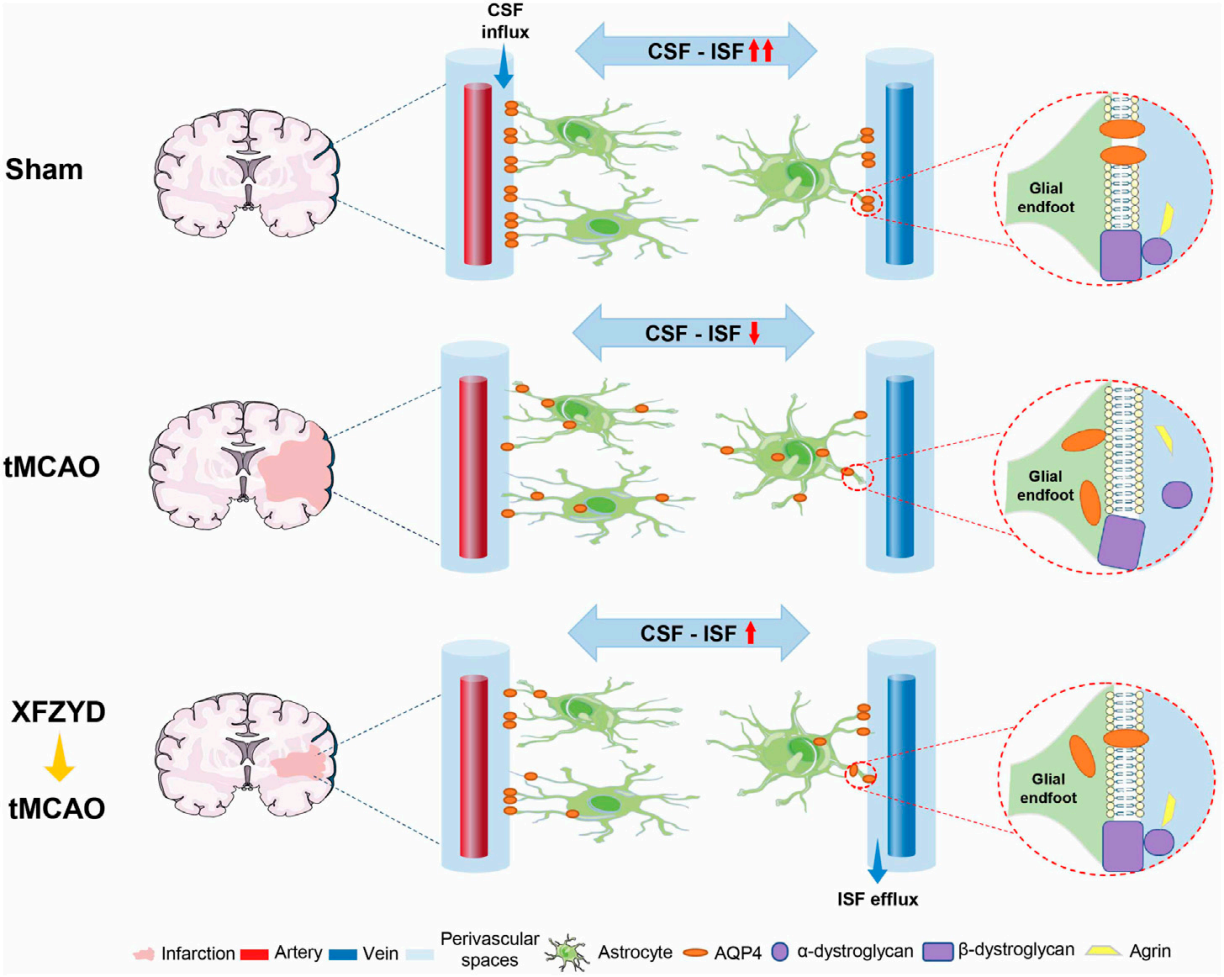
Schematic diagram of pharmacological mechanisms of XFZYD pretreatment for neurological deficits in tMCAO mice. XFZYD pretreatment increased colocalization of AQP4 with its anchoring proteins, α-dystroglycan, β-dystroglycan, and Agrin, to enhance AQP4 polarization on the glial endfoot and exchange efficiency of CSF and ISF in the GS. Thus, XFZYD pretreatment can prevent the onset of neurodegeneration in IS.

In this study, we demonstrated that XFZYD pretreatment represents a novel and effective therapeutic approach that can attenuate neuropathological damage after tMCAO, as supported by reductions in infarct area and the reversal of pathological abnormalities. Our findings also indicated that such improvements may be mediated by the colocalization of AQP4 anchoring proteins to enhance AQP4 polarization on the surface of astrocytes. This hinders glymphatic dysfunction upon ischemia injury and may therefore be helpful for the prevention and treatment of IS (Figure 7).

The GS is a newly discovered metabolic waste clearance system that maintains a delicate balance by promoting the bulk flow of CSF into the parenchyma to exchange with ISF, and by clearing metabolic waste (Iliff et al., 2012). Previous studies reported that the circulation of CSF was impaired after IS and that the GS was involved in the pathology of stroke (Gaberel et al., 2014; Mestre et al., 2020; Lv et al., 2021). These findings are in line with those of the present study. We found that the intensity and distribution area of FITC-D3 decreased significantly after tMCAO; however, these effects were rescued by pretreatment with XFZYD. Altogether, our findings suggest that XFZYD pretreatment may serve as a neuroprotective agent against brain injury after IS by improving glymphatic function.

The impairment of glymphatic function is regulated by astroglia water channel AQP4, which is known to play a key role in the glymphatic pathway (Iliff et al., 2012). AQP4 is expressed exclusively in astrocytes, with a highly polarized distribution in the vascular endfeet. When AQP4 is depolarized, the expression of AQP4 becomes mislocalized and widely distributed in the astrocytes instead of concentrating in the surrounding blood vessels to transport fluid (Nagelhus and Ottersen, 2013). Therefore, the high polarity of AQP4 plays a pivotal role in the influx and efflux of the glymphatic pathway (Mestre et al., 2018). Notably, the decreased polarity of AQP4 expression is concomitant with a significant suppression of glymphatic function after stroke (Back et al., 2020; Lyu et al., 2021a). This may be the reason for the higher mortality rate and more severe neurological deficits in AQP4 knockout (AQP4 (−/−)) mice after IS (Zeng et al., 2012). Consistent with previous studies, our results showed that the expression and polarity of AQP4 were corrected following pretreatment with XFZYD; this may have contributed to the neuroprotection and the recovery of glymphatic function. Notably, AQP4 is complicated to allow bidirectional water flow. Several studies have shown that the up-regulation of AQP4 following ischemic stroke increases cytotoxic edema at early stage, but other studies reported downregulation, which is consistent with our study (Frydenlund et al., 2006; Friedman et al., 2009; Steiner et al., 2012). The discrepancy may be due to several aspects. On the one hand, the severity and region of ischemic stroke are different. An increased expression level of AQP4 was observed after permanent MCAO (Liu et al., 2019). While another study showed that perivascular AQP4 expression was lost in the striatal core following transient ischemia (Frydenlund et al., 2006). On the other hand, the molecular chain of anchoring proteins linked to basement membrane (BM) is broken after tMCAO. Substantial evidence suggests that lack or reduced expression of β-DG and agrin are accompanied by loss of polarized AQP4 expression and localization to astrocytic endfeet (Warth et al., 2004; Noell et al., 2011). Impairment of binding from DG to agrin embedded in the subjacent BM prevented localization of AQP4 to astrocyte endfeet after tMCAO, which is consistent with us (Steiner et al., 2012).

AQP4-anchoring proteins are essential for AQP4 polarization. Mice that are deficient in α-DG, β-DG or agrin show reduced perivascular AQP4 expression in the glial endfeet surrounding blood vessels (Fallier-Becker et al., 2011; Noell et al., 2011; Rauch et al., 2011). The mechanistic action of AQP4 polarization may be due to interaction with DAPC to form a large molecular chain’ changing the expression and location of any member of the molecular chain could affect the polarity distribution of AQP4 (Simon et al., 2018). This notion is consistent with observations in other experimental models of bacterial meningitis which noted that the loss of AQP4 polarity is accompanied by β-DG degradation (Gao et al., 2020). Mice with absent or reduced expression of agrin in the brain showed reduced polarized localization of β-DG and AQP4 at the astrocytic endfeet following tMCAO (Steiner et al., 2012; Amtul et al., 2019); these findings are consistent with the those of the present study. Our data demonstrated that α-DG, β-DG, agrin and AQP4 all exhibited a similar distribution pattern. These proteins colocalize with AQP4 channel aggregates (Guadagno and Moukhles, 2004; Nico et al., 2010). However, the DAPC complex is disrupted by the destabilization of the astrocyte membrane after cerebral ischemia and the colocalization of AQP4 with α-DG, β-DG and agrin become weaker, thus suggesting that AQP4 depolarization might lead to the impairment of glymphatic function following the disruption of stability in the astrocyte endfeet membranes; however, these effects were rescued by pretreatment with XFZYD. Meanwhile, we found that the enhanced tracer influx in the cortex was in accordance with the improvement of colocalization of anchoring proteins in the meninges after XFZYD pretreatment, suggesting that colocalization of AQP4 with α-DG, β-DG, and agrin in the GS may be the potential pharmacological mechanisms of XFZYD pretreatment. Interestingly, the expression profiles of most anchoring proteins are consistent with AQP4 with the exception of α-DG, perhaps due to the tight connection complex caused α-DG to be “hidden” under normal conditions. The separation of glial and vascular basal laminae following lesions cause α-DG epitopes to be “uncovered”; further research is needed to investigate the enhanced expression of α-DG after tMCAO. Moreover, some colocalization coefficient values on tMCAO group are above 0.5 perhaps due to the decreasing trend is not obvious and the measuring of Manders’ overlap coefficient is complicated, thus still remains debated. Some researchers have suggested that the Pearson correlation coefficient is superior to the Manders’ overlap coefficient (Adler and Parmryd, 2010).

Stroke is extremely harmful to human health. Previous studies have shown that preconditioning stimuli had a neuroprotective effect, which has been referred as “cerebral ischemic tolerance” (Kitagawa et al., 1990). Interestingly, a series of subsequent studies have found that in addition to ischemic preconditioning, other drug and non-drug cerebral ischemia preconditioning also can benefit patients with severe cerebral ischemia events (Chang et al., 2018; Han et al., 2019; Zhang et al., 2020a). XFZYD is a well-known traditional Chinese herbal medicine, which plays a protective role in prevention and treatment of various cardio-cerebrovascular diseases, such as angina, myocardial ischemic injury and post-stroke neurological damage (Lee et al., 2011; Shi et al., 2017; Meng et al., 2018; Li et al., 2021). XFZYD contains herbs that have multi-potent properties with significant anticoagulation and anti-inflammation effects. These herbs play an important role in improving blood supply, stimulating blood circulation and controlling the recurrence of thrombosis (Xing et al., 2016; Yeh et al., 2017; Xu et al., 2022). However, the mechanisms underlying these preconditioning effects have yet to be fully elucidated. We hypothesized that pretreatment with XFZYD could promote the recovery of neurological function through the GS in a mouse model of IS. Our results showed that XFZYD alleviated glymphatic dysfunction following stroke by ameliorating AQP4 polarity and anchoring proteins. In addition, synaptogenesis is known to be a crucial and beneficial factor during post-stroke recovery. Agrin is a proteoglycan that is involved in synaptogenesis during the development of the central nervous system (Zhang et al., 2020b). In the present study, XFZYD pretreatment increased the expression levels of agrin; it is possible that this the reason why XFZYD pretreatment reduced nerve damage and accelerated synaptic recovery. In support of this, Lin et al. recently demonstrated that XFZYD improved neurological dysfunction by increasing the expression of synapsin (Zhu et al., 2018), as found in the present study. Moreover, tPA is essential for thrombus breakdown in acute IS but has a strict therapeutic window; XFZYD may represent an adjuvant therapy for tPA in the treatment of IS (Shen et al., 2015; Cheng et al., 2018). A previous study showed that a deficiency of tPA in the brain might reduce glymphatic function by accumulating toxins in the brain (Yu et al., 2019). Therefore, we hypothesize that XFZYD can alleviate this dysfunction; further studies are needed to investigate the relative effects of XFZYD and tPA on GS.

There are several limitations to the current study that need to be considered. In this study, we used *ex vivo* experiments to demonstrate that XFZYD could improve the distribution of CSF influx. It was highly beneficial to explore glymphatic function in different regions in the brain. However, our present study lacked real-time and dynamic flow investigations of the GS. Glymphatic function could also be clinically determined by dynamic contrast enhanced magnetic resonance imaging techniques. Therefore, in future studies, we incorporate *in vivo* two-photon imaging and conduct clinical research to investigate the therapeutic mechanisms of XFZYD for the treatment of stroke. In addition, we did not monitor cerebral blood flow after tMCAO owing to laboratory equipment limitations, which we would add it in the future study. Furthermore, brain edema is also a common feature after ischemic stroke, which largely increased death and disability. In glymphatic system, AQP4 is positive to facilitate the CSF flow into the brain parenchyma, where it mixes with the ISF to eliminate brain wastes and maintain the balance of water in our brain. However, this is inconsistent with cytotoxic edema at early stage. Large evidence indicated that AQP4 may has opposing roles in vasogenic edema when compared to cytotoxic edema. Thus, we will set more time points across the course of cerebral ischemia to better dynamically access the effects of XFZYD *via* regulation of AQP4 in the future research and AQP4 knockout mice could be further used to validate the importance of AQP4 and its anchoring proteins.

## Conclusion

In conclusion, our present study revealed the novel mechanisms by which XFZYD pretreatment alleviates ischemia-reperfusion brain injury in a mouse model. We hypothesize that XFZYD pretreatment targeting AQP4 localization to the perivascular endfeet by stabilizing colocalization of AQP4-anchoring proteins may offer an important approach to enhance glymphatic function and prevent the onset of neurodegeneration in IS.

## Data Availability

The original contributions presented in the study are included in the article/Supplementary Material, further inquiries can be directed to the corresponding authors.
